# Integrated Analysis of Transcriptome Expression Profiles Reveals miRNA-326–NKX3.2-Regulated Porcine Chondrocyte Differentiation

**DOI:** 10.3390/ijms24087257

**Published:** 2023-04-14

**Authors:** Qiao Xu, Yabiao Luo, Zhe Chao, Jibin Zhang, Ximing Liu, Qiguo Tang, Kejun Wang, Shuyi Tan, Meiying Fang

**Affiliations:** 1 Department of Animal Genetics and Breeding, National Engineering Laboratory for Animal Breeding, MOA Laboratory of Animal Genetics and Breeding, Beijing Key Laboratory for Animal Genetic Improvement, College of Animal Science and Technology, China Agricultural University, Beijing 100193, China; 2 Institute of Animal Sciences and Veterinary, Hainan Academy of Agricultural Sciences, Haikou 571100, China; 3 Department of Molecular and Cellular Biology, Beckman Research Institute, City of Hope, Duarte, CA 91006, USA

**Keywords:** pig, vertebral column, RNA-seq, non-coding RNAs, NKX3.2

## Abstract

The porcine body length trait is an essential factor affecting meat production and reproductive performance. It is evident that the development/lengthening of individual vertebrae is one of the main reasons for increases in body length; however, the underlying molecular mechanism remains unclear. In this study, RNA-seq analysis was used to profile the transcriptome (lncRNA, mRNA, and miRNA) of the thoracic intervertebral cartilage (TIC) at two time points (1 and 4 months) during vertebral column development in Yorkshire (Y) and Wuzhishan pigs (W). There were four groups: 1- (Y1) and 4-month-old (Y4) Yorkshire pigs and 1- (W1) and 4-month-old (W4) Wuzhishan pigs. In total, 161, 275, 86, and 126 differentially expressed (DE) lncRNAs, 1478, 2643, 404, and 750 DE genes (DEGs), and 74,51, 34, and 23 DE miRNAs (DE miRNAs) were identified in the Y4 vs. Y1, W4 vs. W1, Y4 vs. W4, and Y1 vs. W1 comparisons, respectively. Functional analysis of these DE transcripts (DETs) demonstrated that they had participated in various biological processes, such as cellular component organization or biogenesis, the developmental process, the metabolic process, bone development, and cartilage development. The crucial bone development-related candidate genes NK3 Homeobox 2 (*NKX3.2*), Wnt ligand secretion mediator (*WLS*), gremlin 1 (*GREM1*), fibroblast growth factor receptor 3 (*FGFR3*), hematopoietically expressed homeobox (*HHEX*), (collagen type XI alpha 1 chain (*COL11A1*), and Wnt Family Member 16 (*WNT16*)) were further identified by functional analysis. Moreover, lncRNA, miRNA, and gene interaction networks were constructed; a total of 55 lncRNAs, 6 miRNAs, and 7 genes formed lncRNA–gene, miRNA–gene, and lncRNA–miRNA–gene pairs, respectively. The aim was to demonstrate that coding and non-coding genes may co-regulate porcine spine development through interaction networks. *NKX3.2* was identified as being specifically expressed in cartilage tissues, and it delayed chondrocyte differentiation. miRNA-326 regulated chondrocyte differentiation by targeting *NKX3.2*. The present study provides the first non-coding RNA and gene expression profiles in the porcine TIC, constructs the lncRNA–miRNA–gene interaction networks, and confirms the function of *NKX3.2* in vertebral column development. These findings contribute to the understanding of the potential molecular mechanisms regulating pig vertebral column development. They expand our knowledge about the differences in body length between different pig species and provide a foundation for future studies.

## 1. Introduction

The evolution of the vertebrate body length has primarily been achieved by changes in the axial skeleton. In many instances, body proportions are reflected by the number of vertebrae per region and the morphology of the vertebrae [1,2,3,4]. For example, body elongation is achieved by increasing the number of vertebrae, the elongation of individual vertebrae, or a combination of both [5]. In mammals, the vertebral formula shows developmental constraint [6]. The number of specific vertebrae in most mammalian species is relatively conserved, but domestic pigs are among the few mammals where variation exists with regard to the number of vertebrae. Several studies have reported quantitative trait loci (QTL) for the number of vertebrae in pigs by genome scan [7,8,9,10,11,12]. Two major QTL have been discovered in these studies, and causative genetic variation has been identified in germ cell nuclear factor (*NR6A1*) [8] and Vertnin (*VRTN*) [9]. However, research on how body elongation is achieved by extending individual vertebrae during vertebral column development remains scarce.

Two distinct developmental processes form bone in mammals. (1) Intramembranous ossification is the developmental process of craniofacial skeletal elements. (2) Endochondral ossification is the developmental process of most of the skeletal elements in the body [13]. The vertebral column is formed through endochondral ossification. The development of the vertebral column is a complex process involving the mesenchymal condensation of undifferentiated cells, hyperplasic and hypertrophic growth, and the mineralization of chondrocytes. The proliferation and hypertrophy of chondrocytes are the main driving forces of vertebral elongation. This process is tightly regulated by various factors, including transcription factors (SRY-box transcription factor 9 (*SOX9*) [14], RUNX family transcription factor 2 (*RUNX2*) [15], and *NKX3.2* [16]), fibroblast growth factors (FGFs) [17], bone morphogenetic proteins (BMPs) [18], and the Wnt [19] or Notch [20] signaling pathway. Defects in these factors often lead to skeletal dysplasia and short stature.

The transcribed genome makes up 70–90% of the whole genome in mammals; only around 2% of it produces proteins. Among the non-coding RNAs (ncRNAs) that lack protein-coding potential, miRNAs are a class of small endogenous ncRNAs (18–24 nt) that regulate gene expressions by the translational repression or degradation of their targets. There is growing evidence that miRNA-mediated mechanisms also play critical roles in bone development [21,22]. For example, miR-196 has been shown to target multiple Hox genes, making it potentially important for the development of vertebrae and the axial skeleton [23]. The loss of miR-140 in mice causes growth defects in the endochondral bones, resulting in dwarfism [24]. miR-140-5p regulates zebrafish embryonic bone development via targeting *BMP-2* [25]. LncRNAs are another class of regulatory ncRNAs, with sizes ranging from 200 bp to 100 kb [26]. The evidence suggests that lncRNAs serve, by various mechanisms, as versatile regulators of diverse aspects of biology. The involvement of lncRNAs in the differentiation of mesenchymal stem cells into osteoblasts has been revealed [27]. Although an increasing number of lncRNAs are being characterized by high-throughput sequencing studies, the regulatory mechanisms involving these lncRNAs during the bone development of pigs or other species have rarely been described.

This study analyzed the non-coding RNA and mRNA expression profiles obtained through next-generation RNA sequencing (RNA-seq) in the TIC of two pig breeds at 1 and 4 months. The functions and interactions between *NKX3.2* and miRNA-326 were verified in chondrocyte models in vitro. This study aimed to obtain deep insights into coding and non-coding RNA expression regulation in the TIC associated with vertebral column development in pigs and to identify the specific mechanisms underpinning the regulation of vertebral lengthening. Furthermore, one advantage of this work is that we investigated for the first time the lncRNA, miRNA, and gene crosstalk that mediates the vertebral column development in pigs.

## 2. Results

### 2.1. Vertebral Development in Pigs

**Figure 1:**
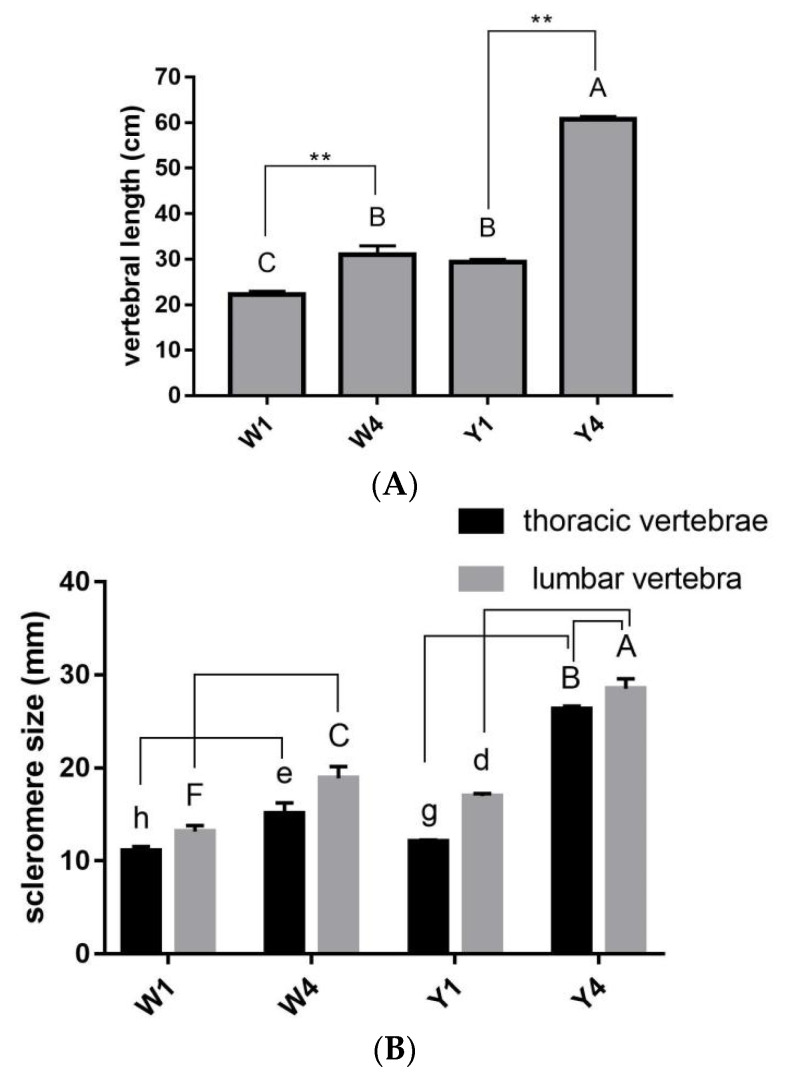
The vertebral development in pigs. Shorter vertebral (**A**), thoracic vertebral, and lumbar vertebral scleromeres (**B**) were detected in the 1-month-old Yorkshire and Wuzhishan pigs than in the 4-month-old pigs. Results are shown as means ± SDs of triplicate measurements. Different letters indicate significant differences (d, e, g, h indicate *p* < 0.05; A, B, C, F indicate *p* < 0.01); the same letters indicate no significant difference. Y1, 1-month-old Yorkshire pigs; Y4, 4-month-old Yorkshire pigs; W1, 1-month-old Wuzhishan pigs; W4, 1-month-old Wuzhishan pigs. ** *p* < 0.01.

The vertebral length and scleromere size of the thoracic and lumbar vertebrae were measured in Yorkshire and Wuzhishan pigs after slaughter. As the pigs grew, the vertebral length of the 4-month-old pigs was significantly longer than that of the 1-month-old pigs, in both breeds (Y4 vs. Y1, W4 vs. W1, *p* < 0.01, Figure 1A). However, the Yorkshire pigs showed much faster growth, and thus, ended up with much longer spines than the Wuzhishan pigs by the age of 4 months (Y4 vs. W4, Figure 1A). The scleromere size of the thoracic and lumbar vertebrae also showed the same pattern, with faster growth in the Yorkshire than in the Wuzhishan pigs (Y4 vs. Y1, W4 vs. W1, *p* < 0.01, Figure 1B). Interestingly, it was also observed that the thoracic vertebrae were always significantly shorter than the lumbar vertebrae in both pig breeds (lumbar vertebrae vs. thoracic vertebrae, *p* < 0.01, Figure 1B). Moreover, frozen sections were made in order to observe the morphology of the chondrocytes in the TIC (Figure S1). Larger hypertrophic chondrocytes were observed in Y4, which is consistent with the results described above.

### 2.2. RNA-Seq Analysis Identifies DETs in the TIC

**Figure 2:**
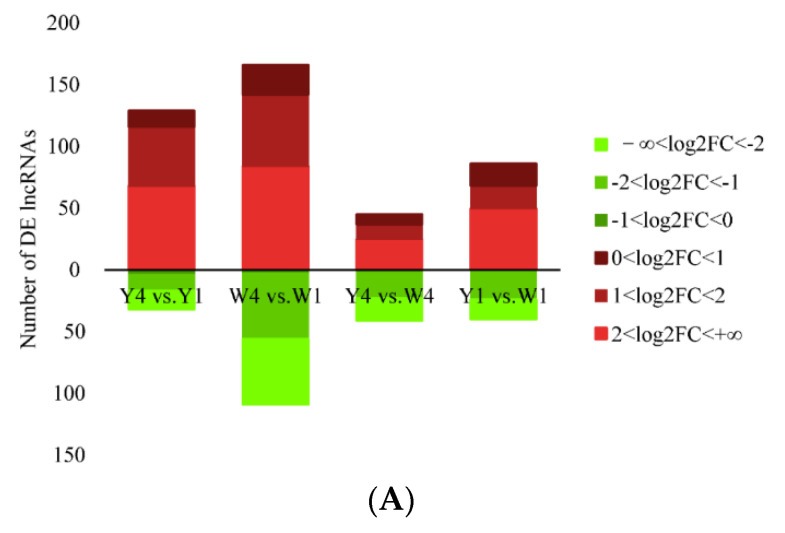

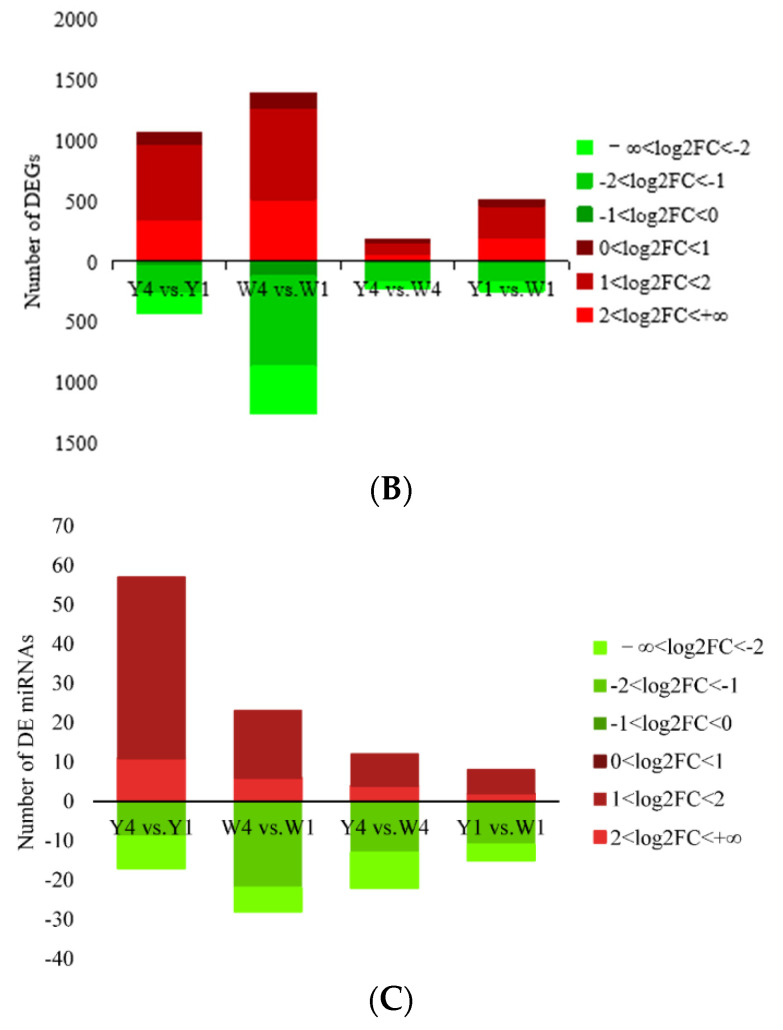
Number of differentially expressed lncRNAs, genes, and miRNAs in the TIC. Number of DE lncRNAs (**A**), DEGs (**B**), and DE miRNAs (**C**) for Y4 vs. Y1, W4 vs. W1, Y4 vs. W1, and Y1 vs. W1 comparisons, respectively. Up-regulated and down-regulated DEGs are represented in red color and green color, respectively. The different ranges of Log2FC are shown by the different color shades.

Among the 89–114 million raw reads from the RNA sequencing, 97% were kept after trimming and filtering, and approximately 80% of the clean reads were mapped to the reference genome (Table 1). With the read counts mapped to each gene, the correlations of the gene expressions among the different individuals were analyzed. The gene expressions among the three biological replicates in each group showed relatively high correlation coefficients (0.845–0.949), suggesting a high similarity of gene expression between the pigs in the same breed at the same age (Figure S2). In addition, the correlation of the gene expression between the two species at the same age was also higher than that between the two ages within the same breed, indicating that age has a more significant effect than breed on gene expression regulation. After strict filtering (Figure S3A) and removal of the potential coding transcripts identified using the coding–non-coding index (CNCI), coding potential calculator (CPC), Pfam scan (PFAM), and phylogenetic codon substitution frequency (PhyloCSF) (Figure S3B), 1735 lncRNAs were obtained. After differential expression analysis, 161 DE lncRNAs were detected in the TIC of the Yorkshire pigs, among which 119 were up-regulated and 42 were down-regulated in Y4 compared to Y1. A total of 275 DE lncRNAs were identified in the TIC of the Wuzhishan pigs, with 148 up-regulated genes and 127 down-regulated genes in W4 compared to W1. Eighty-six DE lncRNAs were detected in the TIC of the 4-month-old pigs in both breeds, including fifty-eight that were up-regulated and twenty-eight that were down-regulated in Y4 compared to W4. One hundred and twenty-six DE lncRNAs were identified in the TIC of the 1-month-old pigs in both breeds, including fifty that were up-regulated and seventy-six that were down-regulated in Y1 compared to W1 (Figure 2A).

In the Yorkshire pigs, 1478 DEGs were identified, among which 1051 genes showed higher expression and 427 genes showed lower expression in Y4 than in Y1. A total of 2643 DEGs, including 1365 with relatively higher and 1278 with relatively lower expression, were identified in the TIC of the Wuzhishan pigs in W4 compared to W1. A total of 404 DEGs were detected in the TIC of the 4-month-old pigs in both breeds, with 245 relatively higher and 159 relatively lower expression genes in Y4 than in W4. A total of 750 DEGs were seen in the TIC of the 1-month-old pigs in both breeds, including 253 somewhat higher and 497 relatively lower expressions in Y1 than in W1 (Figure 2B).

Y4 vs. Y1 revealed 74 DE miRNAs in the TIC, among which 57 were up-regulated and 17 were down-regulated. Fifty-one DE miRNAs, including twenty-three that were up-regulated and twenty-eight that were down-regulated, were detected in the TIC based on W4 vs. W1. Thirty-four DE miRNAs were identified in the TIC of the 4-month-old pigs in both breeds, with twenty-two up-regulated and twelve down-regulated genes based on Y4 vs. W4. Y1 vs. W1 also revealed 23 DE miRNAs in the TIC of the 1-month-old pigs in both breeds, including 15 that were up-regulated and 8 that were down-regulated (Figure 2C).

Further comparison of Y4 vs. Y1 and W4 vs. W1 showed that 73 and 187 DE lncRNAs, 495 and 1660 DEGs, and 50 and 27 DE miRNAs were unique to Y4 vs. Y1 and W4 vs. W1, respectively. Additionally, 88 DE lncRNAs, 983 DEGs, and 24 DE miRNAs were shared between the two contrasts (Figure S4A). Interestingly, among the shared DE lncRNAs, DEGs, and DE miRNAs, one DE lncRNA, five DEGs, and two DE miRNAs were up-regulated in Y4 vs. Y1 and down-regulated in W4 vs. W1, while three DEGs and one DE miRNA were up-regulated in W4 vs. W1 and down-regulated in Y4 vs. Y1.

The comparison of Y4 vs. W4 and Y1 vs. W1 revealed 67 and 107 DE lncRNAs, 301 and 647 DEGs, and 23 and 12 DE miRNAs unique to Y4 vs. W4 and Y1 vs. W1, respectively. In addition, 19 DE lncRNAs, 103 DEGs, and 11 DE miRNAs were shared between the two contrasts (Figure S4B). Interestingly, among the shared DE lncRNAs, DEGs, and DE miRNAs, there were 3 DE lncRNAs, 17 DEGs, and 1 DE miRNA that were up-regulated in Y4 vs. W4 and down-regulated in Y1 vs. W1, while 4 DEGs and 4 DE miRNAs were up-regulated in Y1 vs. W1 and down-regulated in Y4 vs. W4. Subsequently, these DE lncRNAs, DEGs, and DE miRNAs in the TIC based on Y4 vs. Y1, W4 vs. W1, Y4 vs. and W4, and Y1 vs. W1 were integrated into the analysis to reveal the potential regulatory pathways in vertebral column development.

### 2.3. Functional Analysis of DETs

To better understand the biological function of the non-coding RNAs, their target genes were predicted. Previous studies have shown that lncRNAs regulate the expression of neighboring protein-coding genes through cis-acting mechanisms [28,29]. In addition, lncRNAs can also regulate the expression of genes on other chromosomes by trans-acting mechanisms [30,31]. In the comparison groups of Y4 vs. Y1 and W4 vs. W1, 958, 2736, and 1785 genes were predicted by bioinformatic analysis to be the targets of 73 unique DE lncRNAs in Y4 vs. Y1; 187 unique DE lncRNAs in W4 vs. W1; and 88 shared DE lncRNAs in Y4 vs. Y1 and W4 vs. W1, respectively, including 157, 433, and 182 cis-target genes and 820, 2303, and 1658 trans-target genes, of which 19, 86, and 55 genes were regulated by both cis-acting and trans-acting mechanisms. For the 50 unique DE miRNAs in Y4 vs. Y1, 27 unique DE miRNAs in W4 vs. W1, 24 shared DE miRNAs in Y4 vs. Y1 and W4 vs. W1, and 5908, 5381, and 4914 target genes were predicted by miRanda for the 43, 10, and 12 miRNAs that were more abundant in the 4-month-old pigs than in the 1-month-old pigs, and 3148, 4058, and 3253 target genes were predicted for the 7, 17, and 9 miRNAs that were less abundant in the 4-month-old pigs than in the 1-month-old pigs. In addition, 711 and 470 target genes were predicted for two DE miRNAs (up-regulated in Y4 vs. Y1 and down-regulated in W4 vs. W1) and one DE miRNA (up-regulated in W4 vs. W1 and down-regulated in Y4 vs. Y1), which came from 24 shared DE miRNAs in Y4 vs. Y1 and W4 vs. W1.

In the comparison groups of Y4 vs. W4 and Y1 vs. W1, 828, 1214, and 219 genes were predicted by bioinformatics analysis to be the targets of 67 unique DE lncRNAs in Y4 vs. W4; 107 unique DE lncRNAs in Y1 vs. W1; and 19 shared DE lncRNAs in Y4 vs. W4 and Y1 vs. W1, respectively, including 147, 189, and 58 cis-target genes and 693, 1051, and 164 trans-target genes, of which 12, 26, and 3 genes were regulated by both cis-acting and trans-acting mechanisms. For the 23 unique DE miRNAs in Y4 vs. W4, 12 unique DE miRNAs in Y1 vs. W1, and 11 shared DE miRNAs in Y4 vs. W4 and Y1 vs. W1, 4185, 4920, and 2758 target genes were predicted by miRanda for the 17, 7, and 4 miRNAs that were more abundant in the 1- and 4-month-old Yorkshire pigs than in the Wuzhishan pigs, and 2477, 2629, and 1180 target genes were predicted for the 6, 5, and 2 miRNAs that were less abundant in the 1- and 4-month-old Yorkshire pigs than in the Wuzhishan pigs. In addition, 235 and 2301 target genes were predicted for one DE miRNA (up-regulated in Y4 vs. W4 and down-regulated in Y1 vs. W1) and four DE miRNAs (up-regulated in Y1 vs. W1 and down-regulated in Y4 vs. W4), which came from 11 shared DE miRNAs in Y4 vs. W4 and Y1 vs. W1, respectively.

GO and KEGG analyses were performed on the identified DEGs and target genes of the DE lncRNAs and DE miRNAs to evaluate the biological functions and potential regulatory pathways in vertebral column development. In the comparison group of Y4 vs. Y1 and W4 vs. W1, 495 (specific to Y4 vs. Y1), 1660 (specific to W4 vs. W1), and 983 (shared in Y4 vs. Y1 and W4 vs. W1) DEGs were assigned to 422, 511, and 784 significant (*p* < 0.05) GO terms. In the comparison groups of Y4 vs. W4 and Y1 vs. W1, 301 (specific to Y4 vs. W4), 647 (specific to Y1 vs. W1), and 103 (shared in Y4 vs. W4 and Y1 vs. W1) DEGs were assigned to 197, 563, and 391 significant (*p* < 0.05) GO terms. These GO terms were mainly concentrated in the biological process and molecular function categories, including system development, spindle elongation, cell differentiation, cartilage development, and bone maturation (Figure S5A). The top GO terms related to bone development are shown in Table 2. The results of the KEGG analysis revealed that the commonly enriched pathways were involved in the cAMP, TGF-beta, and Wnt signaling pathways (Figure S5B).

The GO analysis (*p* < 0.01) suggested that the cis- and trans-target genes of the DE lncRNAs were primarily involved in the regulation of single organism processes, bone development, and bone maturation (Figure S5C). The KEGG pathway analysis indicated that the target genes were involved in Wnt, TGF-beta, and Notch signaling pathways (Figure S5D). The significant GO terms (*p* < 0.01) of the DE miRNA target genes predominantly included protein binding, developmental processes, skeletal system development, ossification, and axis elongation (Figure S5E). The KEGG analysis significantly enriched the TGF-beta, Wnt, and Notch signaling pathways (Figure S5F).

### 2.4. LncRNA, miRNA, and Gene Interaction Network Construction

**Figure 3:**
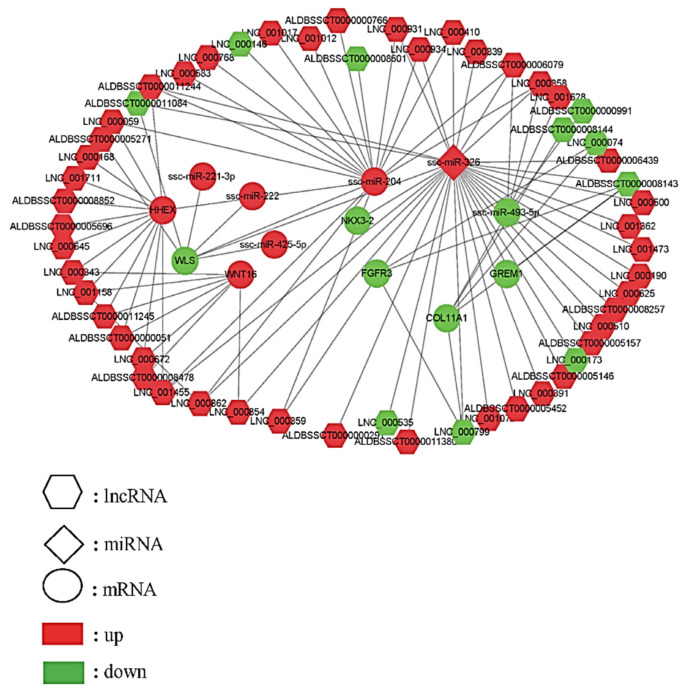
LncRNA, microRNA, and key candidate gene interaction network construction. Hexagonal, prismatic, and circular symbols represent the lncRNA, miRNA, and mRNA, respectively; red represents an up-regulation, and green represents a relative down-regulation compared to the 1-month-old pigs.

To generate an lncRNA, miRNA, and gene interaction network map, the essential genes with functions associated with bone development were identified by integrative GO enrichment analysis with the DEG and non-coding RNA target genes (Table 2). Seven essential candidate genes (*NKX3.2*, *WLS*, *GREM1*, *FGFR3*, *HHEX*, *COL11A1*, and *WNT16*), which were mainly involved in the Wnt and Notch signaling pathway and skeletal development regulation, were further identified by combining functional analysis with literature mining. The interaction network among the lncRNA, miRNA, and essential candidate genes was constructed using Cytoscape v3.2.1 (Figure 3), showing the miRNA–mRNA, lncRNA–mRNA, and lncRNA–miRNA–mRNA interactions. In total, the interaction networks contained 55 lncRNAs (21 annotated and 34 novel lncRNAs), 6 miRNAs, and 7 genes. The results prove that the genes, lncRNAs, and miRNAs were either more or less abundant in the TIC of the Yorkshire and Wuzhishan pigs, based on a comparison of the 4-month-old pigs with the 1-month-old pigs, and that they may contribute together to regulate vertebral column development by the interaction networks.

### 2.5. Quantitative PCR Validation of Differentially Expressed Genes

**Figure 4:**
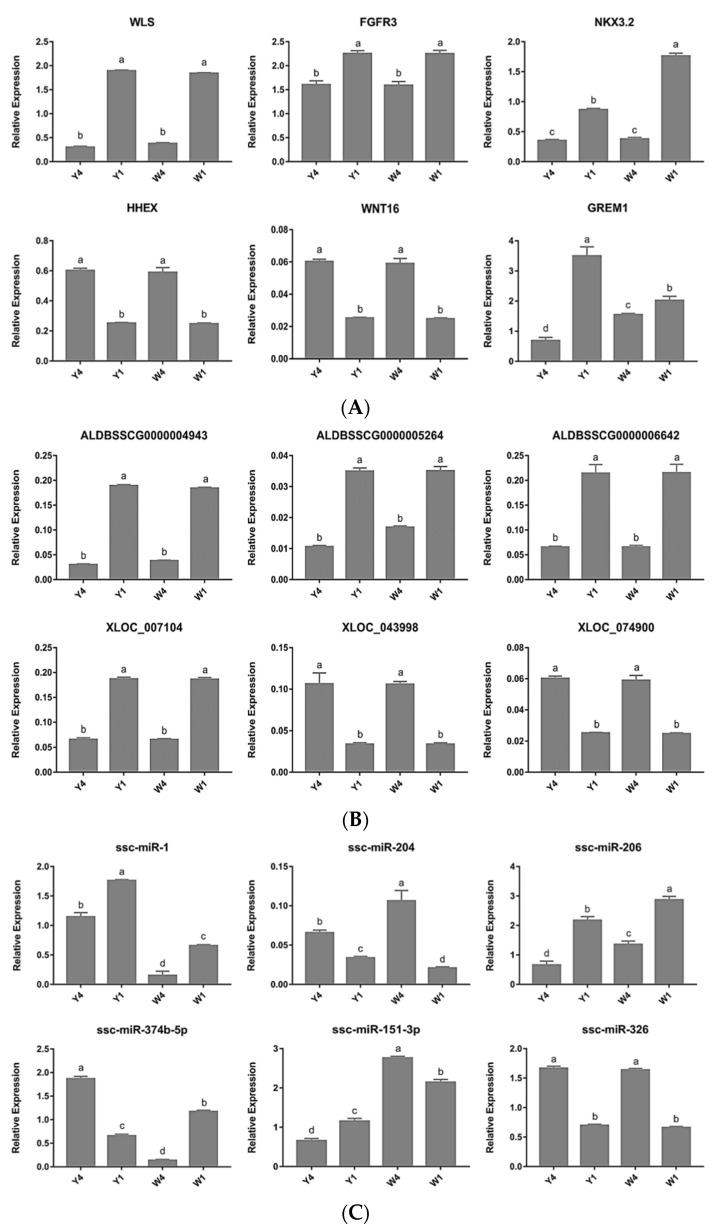
Quantitative PCR-verified RNA-seq results (Y4 vs. Y1; W4 vs. W1; Y4 vs. W1; and Y1 vs. W1). The differentially expressed mRNAs (**A**), lncRNAs (**B**), and microRNAs (**C**) were confirmed by quantitative PCR. Results are shown as means ± SDs of triplicate measurements. Different letters indicate significant differences; the same letters indicate no significant differences. Y1, 1-month-old Yorkshire pigs; Y4, 4-month-old Yorkshire pigs; W1, 1-month-old Wuzhishan pigs; W4, 1-month-old Wuzhishan pigs.

To validate the results of the RNA-seq analysis, six mRNAs, six lncRNAs, and six miRNAs from the TIC were randomly selected and quantified using qPCR (Figure 4). The results confirm that the expression patterns of the DE mRNAs, lncRNAs, and miRNAs were consistent with their expression levels calculated from the RNA-seq data.

### 2.6. NKX3.2 Inhibits Chondrocyte Differentiation

**Figure 5:**
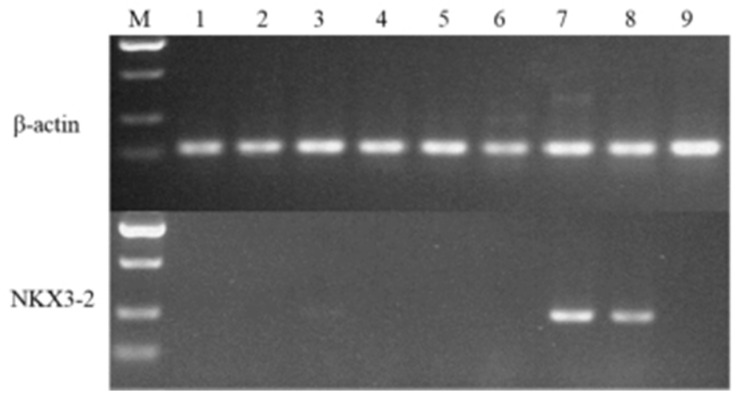
Tissue-specific expression profiles of *NKX3.2* were identified by semi-quantitative reverse transcription PCR (M, maker; 1, heart; 2, liver; 3, spleen; 4, lung; 5, kidney; 6, longissimus dorsi; 7, thoracic intervertebral cartilage; 8, lumbar intervertebral cartilage; 9, bone tissue).

Tissue expression profiles of the key candidate genes were examined in the heart, liver, spleen, lung, kidney, longissimus dorsi, thoracic intervertebral cartilage, lumbar intervertebral cartilage, and bone tissue of the Yorkshire pigs (Figure 5). *NKX3.2* was expressed explicitly in the cartilage tissues, including the thoracic and lumbar intervertebral cartilage, and the band was brighter in the thoracic intervertebral cartilage.

**Figure 6:**
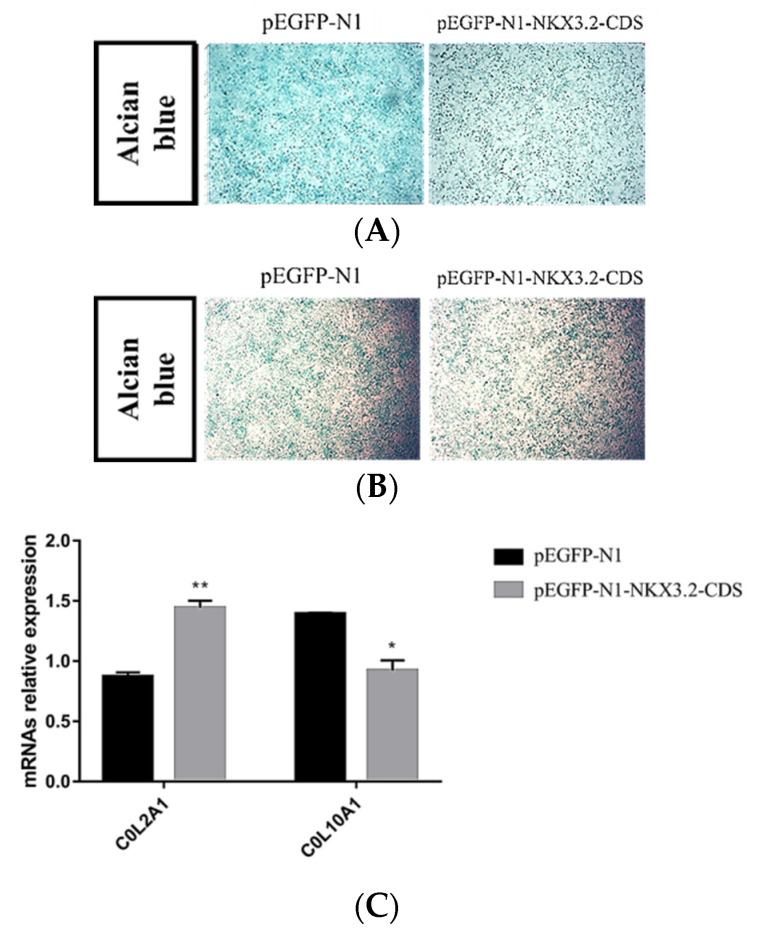

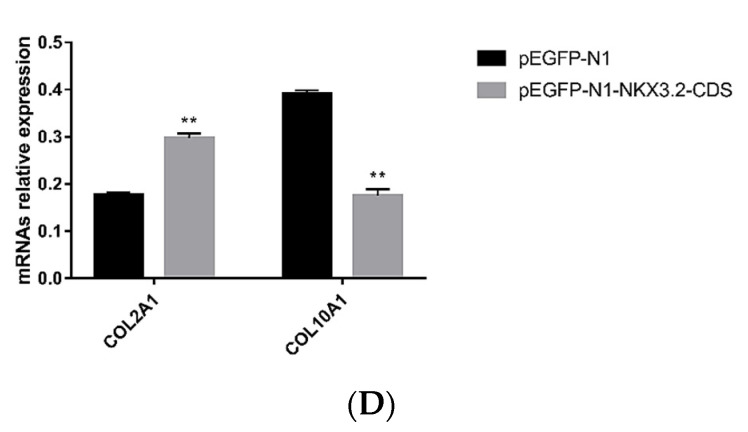
*NKX3.2* inhibits chondrocyte differentiation. ATDC5 cells (**A**,**C**) and pig primary chondrocytes (**B**,**D**) were cultured in chondrogenic differentiation media and treated with control pEGFP-N1 or pEGFP-N1-*NKX3.2*-CDS. After 4 days, the cultures were stained with Alcian blue or harvested for mRNA isolation and qPCR analyses of *COL2A1* and *COL10A1* expressions. Scale bar: 200 um. * *p* < 0.05, ** *p* < 0.01, Student’s *t*-test.

To explore whether *NKX3.2* is involved in chondrocyte development, ATDC5 chondrogenic progenitor cells and pig primary chondrocytes were treated with Lipofectamine 2000 to overexpress *NKX3.2* during culturing in a differentiation medium. Proteoglycan production was reduced by the overexpression of *NKX3.2*, as indicated by a reduction in Alcian blue (Figure 6A,B). Moreover, *NKX3.2* overexpression significantly promoted the gene expression of the chondrocyte marker collagen type II alpha 1 chain (*COL2A1*) (Figure 6C,D), while inhibiting the hypertrophic chondrocyte marker collagen type X alpha 1 chain (*COL10A1*) (Figure 6C,D). Interestingly, the results are consistent in both the ATDC5 cells (Figure 6C) and the pig primary chondrocytes (Figure 6D), suggesting that the overexpression of *NKX3.2* delayed the differentiation of chondrocytes into hypertrophic chondrocytes. Collectively, these data indicate that *NKX3.2* can inhibit chondrocyte hypertrophy.

### 2.7. NKX3.2 Was the Direct Target of miRNA-326

**Figure 7:**
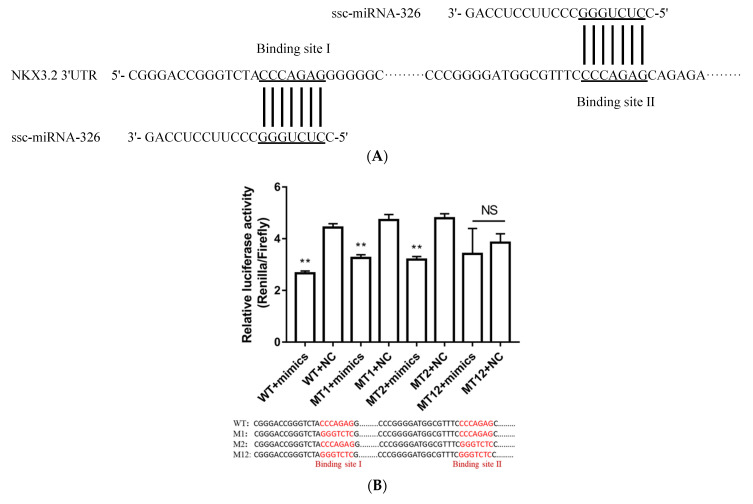
Inhibitory effects of miRNA-326 on *NKX3.2* fragments using the dual-luciferase reporter system. (**A**) The schematic diagram of ssc-miRNA-326 was predicted to bind at the two sites (binding site I and binding site II) in *NKX3.2* 3′UTR. (**B**) The assay was conducted with a fragment spanning the coordinates of binding site I to those of binding site II, in which mutations had been introduced as follows: binding site I and II wild type (WT); binding site I mutation (M1); binding site II mutation (M2); and binding sites I and II mutation (M12). All relative luciferase activity values were normalized to the NC mimics. ** *p* < 0.01; NS, not significant. The underlined and red sequences both indicate the binding sites of miRNA-326 and *NKX3.2*.

The mapping of the lncRNA, miRNA, and gene interaction networks showed that miRNA-326 was predicted to target *NKX3.2* (Figure 3) and had two possible binding sites (binding site I and binding site II) with *NKX3.2* 3′UTR (Figure 7A). To confirm the miRNA-326 target on *NKX3.2*, the binding site I mutation plasmid (MT1), binding site II mutation plasmid (MT2), and double mutations vector in binding site I and binding site II (MT12) were constructed to disrupt putative base pairing between miRNA-326 and *NKX3.2* 3′UTR. The wild-type plasmids of binding site I and binding site II (WT) and MT1 or MT2 or MT12 were co-transfected into HeLa cells with miRNA-326 mimics or NC mimics. The mimics of miRNA-326 significantly decreased the luciferase activity of WT, MT1, and MT2 but did not affect the activity of MT12 (Figure 7B). These results indicate that miRNA-326 could randomly bind binding site I and binding site II of *NKX3.2* 3′UTR.

### 2.8. miRNA-326 Promotes Chondrocyte Differentiation

**Figure 8:**
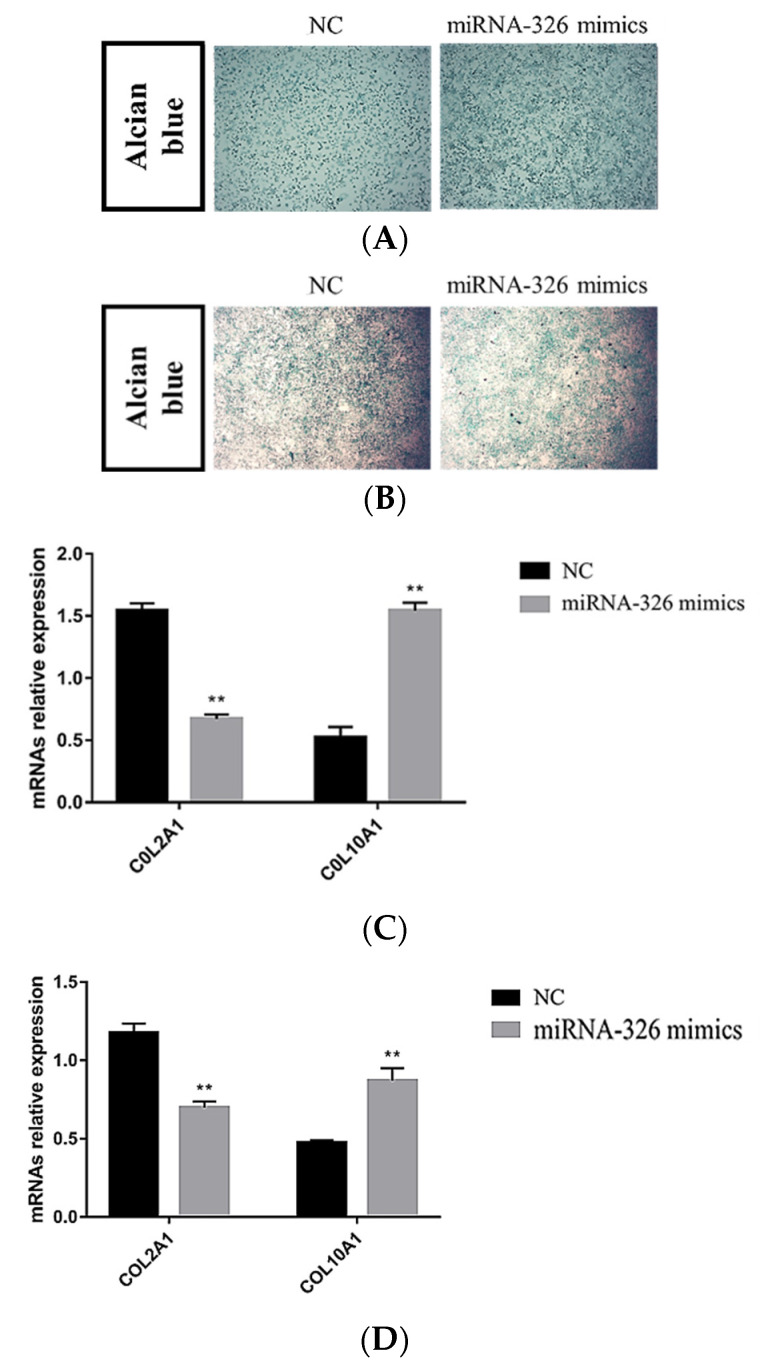
miRNA-326 promotes chondrocyte differentiation. ATDC5 cells (**A**,**C**) and pig primary chondrocytes (**B**,**D**) were cultured in chondrogenic differentiation media and transfected with NC or miRNA-326 mimics. After 4 days, the cultures were stained with Alcian blue or harvested for mRNA isolation and qPCR analyses of *COL2A1* and *COL10A1* and gene expressions. ** *p* < 0.01, Student’s *t*-test.

To determine whether miRNA-326 played a role in the chondrocyte differentiation, ATDC5 chondrogenic progenitor cells and pig primary chondrocytes were transfected with ssc-miRNA-326 mimics during culturing in a differentiation medium. In contrast to the treatment with NC, the overexpression of miRNA-326 resulted in an increase in proteoglycan production (Figure 8A,B) and *COL10A1* expressions (Figure 8C,D) in both the ATDC5 cells and the pig primary chondrocytes. *COL2A1* expression decreased upon miRNA-326 overexpression (Figure 8C,D). Collectively, these data suggest that miRNA-326 can promote chondrocyte hypertrophy.

## 3. Discussion

The vertebral column is a metameric array of tightly interconnected skeletal elements, the vertebrae. It constitutes the supportive yet flexible backbone of all vertebrates. During embryogenesis, the vertebral column is derived from somites, which are primary segments sculpted from the presomitic paraxial mesoderm during somitogenesis [32]. Vertebral column elongation is achieved by somite segmentation and individual vertebra development. In pigs, the differences in body length among different breeds are mainly attributed to the number of thoracolumbar vertebrae, whereas in the same species, the number of vertebrae is fixed, and the changes in body length are primarily caused by the elongation of individual vertebrae. Our results show that the vertebral column of the Yorkshire pigs was significantly longer (*p* < 0.01) than that of the Wuzhishan pigs at the ages of both 1 and 4 months (Figure 1A); this is because Yorkshire pigs have 21 to 23 thoracolumbar vertebrae, while Wuzhishan pigs have 19 thoracolumbar vertebrae, which is mainly due to the lack of thoracic vertebrae. Both pig breeds showed increasing vertebra scleroderma size as they grew (Figure 1B), but the vertebra scleromere in the Yorkshire pigs became much more significant than that in the Wuzhishan pigs by the age of 4 months. Interestingly, the size of the lumbar vertebra scleromere was much longer (*p* < 0.01) than the thoracic vertebra scleromere (Figure 1B) in both pig breeds. Moreover, the frozen sections of TIC also showed larger hypertrophic chondrocytes in Y4. This result is consistent with that described above. To better understand the potential molecular regulation activity underlying this variation of vertebral development, we explored the regulation of gene expression by RNA-seq analysis.

RNA-seq analysis allows for the rapid exploration of the critical genes associated with particular phenotypes or essential biological processes. Although previous RNA-seq studies have shown transcriptome changes in the cartilage of piglet tibia [33], studies on cartilage tissues are still lacking. Therefore, we conducted this RNA-seq study to analyze the non-coding RNAs and mRNA expression profiles in the TIC of the pig. A large number of DEGs that play essential roles in the regulation of bone development were identified. Key candidate genes were further identified through a combination of functional analysis with literature mining, including *NKX3.2* [34,35,36,37,38,39,40], *WLS* [41,42,43], *GREM1* [44,45,46], *FGFR3* [47,48,49,50,51], *HHEX* [52,53,54], *COL11A1* [55,56,57], and *WNT16* [58,59]. These genes were mainly enriched in the BMP, FGF, Wnt, and Notch signaling pathways, suggesting that they are involved in the development of the pig vertebral column. In addition to the coding genes, the target genes of the lncRNAs or miRNAs were also enriched in these pathways.

*WLS* (also known as *GPR177*) is a chaperone protein that is required for the secretion of both canonical and non-canonical Wnt ligands [41]. The loss of *WLS* specificity in cartilage resulted in delayed chondrocyte differentiation, disorganized chondrocyte arrangement, and impaired endochondral bone formation [42]. Recent studies have shown that skeletal homeostasis was altered with decreased bone formation within 1–2 weeks of conditional *WLS* knockout in adult mice (5 months old) [43]. *GREM1* is an antagonist of bone morphogenetic proteins and is expressed in osteoblasts. *GREM1* null mice exhibited developmental skeletal abnormalities, decreased weight and body fat, and shortened femoral length [44]. The latest research suggests that the hyperexpression of *GREM1* specifically blocks chick phalanx development by inhibiting PFR activity [45] and causing severe defects of skeletogenesis in *Trim28*^MKO^ mice [46]. *HHEX*, a homeodomain protein, is a transcription factor that participates in cell proliferation, differentiation, and migration. A study in mouse chondrogenic cell line ATDC5 showed that HHEX protein expression and subcellular localization were associated with chondrocyte maturation [52]. Recent studies have shown that *HHEX* negatively regulates osteoclast differentiation [53]. Our recent study also found that a TA haplotype on the *HHEX* promoter was significantly associated with body length in pigs [54]. *COL11A1* is normally found in cartilage, which is responsible for the formation of cartilage structures in the early stages of skeleton development. Li et al. showed that chondrodysplasia in mice is associated with mutations in *COL11A1* [55]. A novel deleterious heterozygous mutation in *COL11A1* was associated with severe skeletal dysplasia [56]. A novel missense variant (G to A in exon 45) of *COL11A1* resulted in short-limb skeletal dysplasia [57]. *WTN16*, a ligand of the Wnt signaling pathway, is associated with bone density, cortical thickness, bone strength, and fracture risk [58,59]. Wnt16^−/−^ zebrafish exhibited significant deformities in the head, spine, and tail and decreased bone mineral density and trabecular bone [59].

Non-coding RNAs (ncRNAs) have evolved in eukaryotes as epigenetic regulators of gene expression. The most abundant regulatory ncRNAs are miRNAs and lncRNAs. Each class of ncRNAs regulates gene expression through distinct mechanisms [60]. Therefore, to better understand the complex mechanism of vertebral column development, we constructed the lncRNA–mRNA, miRNA–mRNA, and lncRNA–miRNA–mRNA interaction networks for those key DEGs we identified (Figure 3). The lncRNA-target regulatory interaction networks, comprising 55 DE lncRNAs (of which 21 were annotated and 34 were novel) and the essential candidate genes, were constructed (Figure 3). *FGFR3* was predicted to be a putative target of ALDBSSCT0000008143, LNC000074, and LNC000799. *FGFR3* is mainly expressed in cartilage and negatively regulates bone growth [47,48]. A recent study suggested that mice with a conditional knockout of *FGFR3* in the chondrocytes displayed overgrowth of bone with a significantly increased bone mass at both 1 and 4 months of age [49]. A study on postmenopausal osteoporosis mice showed that FGFR3 activation inhibited the ability of bone regeneration and bone mineralization [50]. Human FGFR3-related skeletal dysplasia was also summarized in a recent review [51]. In the present study, *FGFR3* showed a significantly lower expression in the 4-month-old pigs than in the 1-month-old ones (Figure 4A), which is consistent with that reported above. ALDBSSCT0000008143 was predicted to regulate the expression of *FGFR3* via cis-acting mechanisms and was expressed at a lower level in the 4-month-old than in the 1-month-old pigs (Figure 4B). A previous study suggested that lncRNAs could activate the transcription of nearby genes via cis-acting mechanisms by recruiting remodeling factors to local chromatin [61]. Therefore, it is reasonable to speculate that ALDBSSCT0000008143 may target *FGFR3* via cis-acting mechanisms to regulate vertebral column development based on the positive relationship between ALDBSSCT0000008143 and FGFR3 observed in this study.

To understand how the interactions between miRNAs and their target genes regulate vertebral column development, interaction networks between DE miRNAs and key candidate genes were constructed. A total of six miRNAs and seven genes formed eight miRNA–gene pairs. Of the above pairs, the integrated analyses identified anti-correlated ssc-miR-326–*NKX3.2* pairs (Figure 3). *NKX3.2*, also known as Bapx1, is a transcription factor that belongs to the NK family of homeobox genes. It has been suggested that it plays a role in inhibiting chondrocyte hypertrophy and maintaining the chondrocyte phenotype during chondrogenic differentiation [34,35]. In recent years, the mutation and function of *NKX3.2* have been extensively studied in mouse [36] and zebrafish models [37,38], as well as in human [39] skeletal abnormalities. In this study, *NKX3.2* was explicitly expressed in cartilage tissues (Figure 5). This suggests that *NKX3.2* may play an essential role in the development of the porcine vertebral column. The function of *NKX3.2* in inhibiting chondrocyte hypertrophy, and thus, delaying chondrocyte differentiation was further identified in vitro (Figure 6). These results are similar to those of previous studies. Interestingly, in vivo cartilage-specific *NKX3.2* overexpression resulted in overall size reduction and shorter vertebral columns [40].

Furthermore, the targeted relationship between miRNA-326 and *NKX3.2* was demonstrated by a dual-luciferin assay. miR-326 was also further identified to promote chondrocyte hypertrophy by regulating the expression of *NKX3.2* (Figure 7 and Figure 8). Recently, studies have shown that miR-326 acts as a negative modulator for sonic hedgehog (Shh) signaling [62], which is a crucial regulatory pathway for bone morphogenesis [63]. Collectively, these results indicate that the interaction of miR-326 and *NKX3.2* may jointly influence the development of vertebral columns by regulating chondrocyte differentiation. Thus, further investigations into the signaling pathways in which *NKX3.2* is involved in regulating vertebral column development are needed, by ChIP-seq or RNA-seq analysis or a combination of both. The study of this pathway can be applied not only to the breeding of body length traits in pig production, the improvement of pork yield, and an increase in the efficiency of pig production, but it can also provide an ideal small-sized pig model for human physiological and pathological research.

Recent reports have suggested that lncRNAs can potentially interact with miRNAs and then regulate the expression of genes. This study systematically analyzed the complex interactions among miRNAs, lncRNAs, and their target genes and provided lncRNA–miRNA–gene interaction networks. For example, LNC000173 (Gene ID: XLOC_007104) was observed to have a significantly lower expression in the 4-month-old pigs than in the 1-month-old pigs (Figure 4B) and was predicted to compete with miR-326 to bind with NKX3.2. Therefore, it was speculated that LNC000173 might regulate the porcine vertebral column development via the pig’s LNC000173–miR-326–*NKX3.2* interaction. The lncRNA–miRNA–mRNA interaction networks revealed a setting for the competition of endogenous RNAs that was possibly involved in the vertebral column development process. However, this hypothesis requires further study for verification.

## 4. Materials and Methods

### 4.1. Animal Sample Collection and RNA Isolated

Yorkshire pigs at the ages of 1 month (Y1, n = 3) and 4 months (Y4, n = 3) were obtained from the Beijing pig breeding farm (Beijing, China). Wuzhishan pigs at the ages of 1 month (W1, n = 3) and 4 months (W4, n = 3) were obtained from the Animal Sciences and Veterinary Institute, Hainan Academy of Agricultural Sciences (Haikou, China). The TIC was sampled after the animals were slaughtered. The tissue samples were frozen in liquid nitrogen and stored at −80 °C before RNA isolation.

According to the manufacturer’s instructions, the total RNA was isolated from each TIC tissue using TRIZOL^®^ reagent (Invitrogen, Carlsbad, CA, USA). The RNA quality was examined on 1% agarose gel. The RNA purity was checked using the NanoPhotometer^®^ spectrophotometer (Implen, Westlake Village, CA, USA). The RNA concentration was measured using the Qubit^®^ RNA Assay Kit in the Qubit^®^ 2.0 Fluorometer (Life Technologies, Carlsbad, CA, USA). The RNA integrity was assessed using the RNA Nano 6000 Assay Kit in the Bioanalyzer 2100 system (Agilent Technologies, Santa Clara, CA, USA).

### 4.2. Library Preparation for lncRNA Sequencing and Data Analysis

Sequencing libraries were prepared using 3μg of RNA per sample. The samples were indexed with the NEBNext^®^ Ultra™ Directional RNA Library Prep Kit for Illumina (NEB, Ipswich, MA, USA), following a procedure described previously [64]. The libraries were sequenced on a HiSeq 2500 platform (Illumina, San Diego, CA, USA), and 125 bp paired-end reads were generated. Raw reads were processed through in-house Perl scripts to remove reads containing adapter, poly-N, and low-quality reads. The remaining reads were mapped to the porcine reference genome using TopHat (v2.0.9). Mapped reads from each sample were assembled by both Scripture (beta2) [65] and Cufflinks (v2.1.1) [66] in a reference-based approach. The assembled transcripts were evaluated using five criteria to identify lncRNAs: (1) transcripts with exon number ≥2 were retained; (2) transcripts with length > 200 bp were retained; (3) known no-lncRNA annotations were removed; (4) transcripts with fragments per kilobase of exon per million fragments mapped (FPKM) < 0.5 were removed; (5) CNCI (v2) [67], CPC (0.9-r2) [68], PFAM (v1.3) [69], and PhyloCSF (v20121028) [70] were used to distinguish the mRNAs from the lncRNAs (Figure S3A). The transcripts predicted to have coding potential by all four tools described above were excluded, and those without coding potential were classified as lncRNA candidates (Figure S3B). The transcripts excluded above were used as candidate mRNAs. PHAST v1.3 was used for conservation analysis for the coding genes and lncRNAs [71].

Cuffdiff (v2.11) was used to calculate the FPKM of the lncRNAs and coding genes in each sample. Transcripts with a *p*-adjust of <0.05 were assigned as DETs. We searched the coding genes 100 kb upstream and downstream of the lncRNA as the cis-target genes. The potential trans-target genes of the lncRNA were identified by the Pearson correlation coefficients (r > 0.95 or r < −0.95) of their expression levels.

### 4.3. Library Preparation for microRNA Sequencing and Data Analysis

Three μg of RNA per sample was used as the input material for the small RNA library. The sequencing libraries were generated using NEBNext^®^ Multiplex Small RNA Library Prep Set for Illumina^®^ (NEB, Ipswich, MA, USA), following a procedure described previously [64]. The levels of miRNA expression were estimated using transcript per million (TPM) through the following normalization formula: normalized expression = mapped reads/total reads × 1,000,000 [72].

Differential expression analysis of the miRNAs among the groups was performed using the DESeq R package (1.8.3). The *p*-values were adjusted using the Benjamini and Hochberg methods. miRNAs with a corrected *p*-value of < 0.05 were classified as significantly differential expressions.

### 4.4. GO and KEGG Enrichment Analysis

Gene Ontology (GO) and Kyoto encyclopedia of genes and genomes (KEGG) enrichment analyses of the DEGs or target genes of the DE lncRNAs and DE miRNA were conducted as described previously [64]. The GO terms and KEGG pathways with *p*-values less than 0.05 were considered significantly enriched by the DEGs.

### 4.5. Construction of lncRNA, miRNA, and mRNA Regulatory Networks

The lncRNA, miRNA, and mRNA interaction networks were constructed as previously described [62] and visualized using Cytoscape v3.2.1 [73].

### 4.6. Cell Isolation, Culture, and Histological Analysis

The HeLa and ATDC5 cell lines were purchased from Xiehe Medical University (Beijing, China) and Otwo Biotech (Shenzhen, China), respectively, and the primary chondrocytes from the TIC of newborn piglets were isolated as previously described [74]. All the cells were cultured in a medium that contained DMEM, 10% FBS, and 1% penicillin/streptomycin.

For chondrocyte differentiation, the ATDC5 cells and pig primary chondrocytes were grown in a medium containing 1% insulin–transferrin–selenium (ITS, Gibco). To perform Alcian blue staining, the cells were first fixed in 10% NBF for 30 min and then incubated with a 1% Alcian blue/3% acetic acid solution for 30 min. The cultures were then rinsed with 70% ethanol, followed by ddH_2_O, and air-dried before imaging.

For transfection, the ATDC5 cells and pig primary chondrocytes were transfected with 2 µg of the overexpression vectors or empty plasmid using Lipofectamine 2000 (Invitrogen) in each well of a 6-well plate. After being transfected for two days, the cells were harvested for RNA extraction.

For the tissue slices, the cartilage tissue samples from the TIC were embedded in OCT tissue freezing medium (Leica, Germany) and serially sectioned (6 μm thickness). The obtained cartilage tissue sections were observed under a 10 × 20 camera microscope.

### 4.7. Plasmid Construction and Dual-Luciferase Reporter Assay

For the *NKX3.2* overexpression plasmids, the CDS sequence was cloned into the EcoRI and BamHI (NEB, Beverly, MA, USA) restriction sites of the expression vector pEGFP-N1. The 3′UTR of the *NKX3.2* fragment containing the predicted target site was cloned into the psiCHECK2 vector with AsiSI and PmeI (NEB, Beverly, MA, USA). The primer was synthesized by Sangon (Shanghai, China) to introduce the mutation of some bases at the putative binding site of miRNA-326 in *NKX3.2*. All the primers used are listed in Supplementary Table S1.

HeLa cells were seeded into 24-cell plates and transfected 24 h later. The miRNA-326 was co-transfected with 100 ng of psicheck2-*NKX3.2*-fragment and 40 pmol of miRNA-326 mimics or negative control (NC) mimics (RiboBio, Guangzhou, China). Thirty-six hours after transfection, the Firefly and Renilla luciferase activities were measured using the Dual-Glo luciferase kit (Promega). The assays were repeated three times.

### 4.8. Measurement of Gene Expression

Semi-quantitative reverse transcription PCR (SqRT-PCR) was performed for the tissue expression profiles as previously described [75], and *β-actin* was an endogenous control gene.

For validating the differentially expressed mRNAs, lncRNAs, and miRNAs, six lncRNAs, six mRNAs, and six miRNAs (Table 3) were randomly selected. Quantitative PCR (qPCR) was performed using the SYBR Green Master Mix (TIANGEN, Beijing, China) according to a previously described procedure [64]. The relative expression was normalized using the 2^−ΔΔCt^ method with *β-actin* (genes and lncRNAs) and U6 small nuclear RNA (miRNAs) as an internal reference. First, the cycle threshold (CT) values of the target gene were normalized with the CT values of the internal reference gene for all the samples and calibration samples. The calibration sample was a mixture of all the individual sample cDNAs. Second, the ∆CT values of the calibration samples were used to normalize the ∆CT values of the test samples. Finally, the expression-level ratio was calculated.

∆CT(test) = CT(target, test) − CT(ref, test)

∆CT(calibrator) = CT(target, calibrator) − CT(ref, calibrator)

∆∆CT = ∆CT(test) − ∆CT(calibrator)

2−∆∆CT = Relative expression. 

Each biological duplicate consisted of three technical replicates. The miRNA primers were designed and synthesized by RiboBio (Guangzhou, China), who treat primer sequences as confidential information. All the primers used for this section are listed in Supplementary Table S2.

### 4.9. Statistical Analysis

The statistical analysis results are shown as the means ± standard error (SE). The significant differences among the groups were identified through one-way analysis of variance (ANOVA). A comparison of the different groups was made using Duncan’s new multiple range test in the R package agricolae (version 1.2-8), R version 3.3.3. The differences were considered statistically significant at *p*-values < 0.05.

## 5. Conclusions

In this study, we profiled the expressions of lncRNAs, miRNAs, and mRNAs at two-time points of TIC development (1 and 4 months old) in Yorkshire and Wuzhishan pigs. Our analysis suggests that several lncRNAs, miRNAs, and genes are involved in critical biological processes associated with bone development. We also constructed the lncRNA–miRNA–gene interaction networks based on the transcription profiles derived from pig TIC. Moreover, the interaction between miRNA-326 and *NKX3.2* was identified as being involved in the regulation of chondrocyte hypertrophy differentiation. Therefore, our results contribute to the understanding of the underlying molecular mechanisms of pig body length variation and provide new insights and valuable information for the study of body length variations in other animals from the perspective of vertebral column development.

## Figures and Tables

**Table 1:** Summary of reads filtering, mapping, and counting in RNA-seq.

Sample Name	Raw Reads	Clean Reads	Error Rate (%)	Q20 (%)	Q30 (%)	% of Mapped Reads	Uniquely Mapped Reads	Reads Mapped to mRNA
W11	113,505,514	11,044,2434	0.01	97.92	94.59	79.28	72,844,716	21,521,718 (55.61%)
W12	106,805,890	103,934,400	0.01	97.54	93.7	79.25	69,206,807	22,967,413 (62.93%)
W13	108,723,412	105,856,996	0.01	97.79	94.32	79.78	71,235,208	23,608,836 (62.78%)
W41	89,009,068	86,255,042	0.02	97.26	93.11	78.16	59,558,565	20,819,812 (66.35%)
W42	94,091,046	91,527,780	0.01	97.69	94.05	78.16	60,244,736	21,465,873 (67.78%)
W43	103,048,596	100,034,036	0.01	97.39	93.48	76.30	68,714,104	23,544,258 (64.73%)
Y11	98,092,402	95,272,188	0.02	97.35	93.27	81.80	68,345,799	22,528,226 (63.61%)
Y12	99,505,204	96,391,826	0.02	97.01	92.59	80.71	69,699,510	23,019,609 (62.46%)
Y13	102,313,346	99,334,904	0.02	97.18	92.93	80.24	70,930,309	22,432,338 (60.06%)
Y41	103,154,682	100,089,328	0.01	97.41	93.42	81.12	71,909,642	24,156,222 (65.58%)
Y42	95,680,318	93,170,738	0.01	97.65	93.98	80.75	66,502,651	20,678,419 (59.13%)
Y43	101,495,130	98,308,836	0.02	97.31	93.18	79.44	68,162,985	22,480,597 (64.02%)

**Table 2:** Significant GO terms and the functional genes associated with bone development.

GO Term	*p*-Value	Genes
Bone remodeling	6.22 × 10^−7^	*WNT16, RASSF2, RAB3D, PTK2B, RAC3, ADAM8, MEPE, UBASH3B, DCSTAMP, SYK,* *GREM1, INPP5D, PTGER4, BGLAP, CEACAM1*
Bone resorption	6.45 × 10^−5^	*RAB3D, PTK2B, RAC3, ADAM8, UBASH3B, SYK, INPP5D, PTGER4, BGLAP*
Bone maturation	0.000233	*LTF, FGFR3, GREM1, THBS3, RFLNA, RYR1*
Bone development	0.000411	*TYROBP, LTF, FGFR3, PTPRCAP, IMPAD1, PTPN6, CSGALNACT1, FLI1, MATN1, GREM1, THBS3, AKAP13, HAS2, PTGER4, RFLNA, RYR1, BGLAP*
Bone mineralization	0.001976	*LTF, FGFR3, PTK2B, SRGN, RSPO2, MEPE, MATN1, PKDCC, GREM1, RFLNA, BGLAP*
Bone morphogenesis	0.031658	*LTF, FGFR3, IMPAD1, CSGALNACT1, MATN1, THBS3, HAS2*
Cartilage development	0.003859	*SCIN, FGFR3, IMPAD1, NKX3-2, MAPK14, SOX5, CNMD, CSGALNACT1, RSPO2, MATN1, PKDCC, GREM1, THBS3, CYTL1, RFLNA*
Skeletal system development	0.000388	*SCIN, TYROBP, LTF, FGFR3, PTPRCAP, RASSF2, IMPAD1, NKX3-2, PTPN6, MAPK14, MDFI, SLC35D1, MATN3, SOX5, CNMD, HAPLN3, CSGALNACT1, RSPO2, FLI1, MEPE, EXTL1, MATN1, PKDCC, HHIP, GREM1, THBS3, AKAP13, CYTL1, HAS2, PTGER4, SOX11, RFLNA, RYR1, COL11A1, BGLAP*
Axis specification	0.05477	*COBL, MDFI, WLS, STIL, FOXA2, HHEX, BASP1*
Axis elongation	0.693058	*PTK7*
Chondrocyte differentiation	0.001366	*SCIN, FGFR3, IMPAD1, NKX3-2, MAPK14, SOX5, MATN1, PKDCC, GREM1, CYTL1, RFLNA*
Regulation of osteoblast proliferation	0.06172	*LTF, GREM1, HPSE*
Osteoblast differentiation	0.344098	*LTF, RASSF2, RSPO2, NOCT, IL6R, PTGER4, ID4, SOX11, BGLAP*
Osteoclast differentiation	0.005422	*TYROBP, LTF, RASSF2, MAPK14, UBASH3B, DCSTAMPINPP5D, LILRB4, BGLAP*

**Table 3:** Differentially expressed lncRNAs, mRNAs, and microRNAs between different ages in two pig breeds.

Genes	Transcript ID	Gene ID/Name	Y4_vs_Y1_ log2FC	W4_vs_W1_ log2FC	*p*-Adjust
lncRNAs	ALDBSSCT0000008143	ALDBSSCG0000004943	−1.7	−1.8	0.0022
	LNC_001455	XLOC_074900	3.7	3.9	0.0032
	LNC_000862	XLOC_043998	2.7	4.1	0.0012
	LNC_000173	XLOC_007104	−3.6	−5.7	0.0092
	ALDBSSCT0000010956	ALDBSSCG0000006642	−4.6	−3.3	0.0446
	ALDBSSCT0000008644	ALDBSSCG0000005264	−4.7	−3.7	0.0119
mRNAs	ENSSSCG00000024594	*NKX3.2*	−1.7	−2.3	0.0012
	ENSSSCG00000004808	*GREM1*	−1.5	−1.8	0.0206
	ENSSSCG00000003795	*WLS*	−1.2	−1.2	0.0022
	ENSSSCG00000030827	*FGFR3*	−1.9	−1.2	0.0056
	ENSSSCG00000010472	*HHEX*	1.1	1.8	0.0302
	ENSSSCG00000016617	*WNT16*	3.6	4.5	0.0012
microRNAs		ssc-miR−1	−2.1	−2.0	0.0000
		ssc-miR-204	1.7	1.3	0.0012
		ssc-miR-206	−2.0	−2.9	0.0000
		ssc-miR-326	2.6	3.4	0.0098
		ssc-miR-151-3p	−1.5	1.3	0.0000
		ssc-miR-374b-5p	1.4	−1.6	0.0000

## Data Availability

The data are available under project number PRJNA949733.

## Supplementary Materials

The following supporting information can be downloaded at: https://www.mdpi.com/article/10.3390/ijms24087257/s1.
