# Supplementary material for: Integrated Analysis of Transcriptome Expression Profiles Reveals miRNA-326–NKX3.2-Regulated Porcine Chondrocyte Differentiation
Source: Int J Mol Sci. 2023 Apr 14;24(8):7257. doi: 10.3390/ijms24087257 (PMC10138716; doi:10.3390/ijms24087257)
Supplement: Supplementary file 1 [file ijms-24-07257-s001.zip › ijms-2246613-supplementary.pdf]

Table S1. Sequence of primers used to construct plasmid. Yellow mark: enzyme loci; Red mark: binding or mutation site.

Primers	Enzyme	Sequence（5'-3'）
NKX3.2-CDS-F (pig)	EcoRI	ATTGAATTCTGGGCAGCGCAGGAGT
NKX3.2-CDS-R (pig)	BamHI	ATTGGATCCGGGCACAATCTGCATAGCC
NKX3.2-CDS-F (mouse)	EcoRI	ATTGAATTCCTGAGGTCGCCCAGAGCT
NKX3.2-CDS-R (mouse)	BamHI	ATTGGATCCCAAGTCCCAGCAGGATTTCA
NKX3.2-wt1-3'UTR-F	AsiSI	ATTGCGATCGCCCAGGATGCGCGCTCTGGCCTCGCC GTCTACCCAGAGGGGGGCGCCTTTCTCTAACTTGAG CAGTGGCATCTAGGGGCCCTGTTC
NKX3.2-wt1-3'UTR-R	PmeI	TAAGTTTAAACCAAATCCAGCATTGTGGTGGTAATAA ATAACGTCCAAAGGCTTCCGGGAAGGTGAGGGGTGG GGGGAACAGGGCCCCTAGATGCC
NKX3.2-mut1-3'UTR-F	AsiSI	ATTGCGATCGCCCAGGATGCGCGCTCTGGCCTCGCCG TCTAGGGTCTCGGGGGCGCCTTTCTCTAACTTGAGCA GTGGCATCTAGGGGCCCTGTTC
NKX3.2-mut1-3'UTR-R	PmeI	TAAGTTTAAACCAAATCCAGCATTGTGGTGGTAATAA ATAACGTCCAAAGGCTTCCGGGAAGGTGAGGGGTGG GGGGAACAGGGCCCCTAGATGCC
NKX3.2-wt2-3'UTR-F	AsiSI	ATTGCGATCGCATTTGGATTGTGTCTGCCCGGTCGTTT CCCCAGAGCAGAGAGAACTCCTGGAGACCACAAAAG CCTGAATCCCAGAATTTCAGGC
NKX3.2-wt2-3'UTR-R	PmeI	TAAGTTTAAACTACACATAGATTGGTAGCATGGATACCA TGAACTAGTGTGGCCTAGCGCACGAAGCTCCCGGCAGGC CTGAAATTCTGGGATTCA
NKX3.2-mut2-3'UTR-F	PmeI	ATTGCGATCGCATTTGGATTGTGTCTGCCCGGTCGTTTC GGGTCTCCAGAGAGAACTCCTGGAGACCACAAAAGCC TGAATCCCAGAATTTCAGGC
NKX3.2-mut2-3'UTR-R	AsiSI	TAAGTTTAAACTACACATAGATTGGTAGCATGGATACCA TGAACTAGTGTGGCCTAGCGCACGAAGCTCCCGGCAGGC CTGAAATTCTGGGATTCA

Table S2: The primer sequences for SqRT-PCR and qPCR.

Species	Gene	Primers（5'-3'）	Application
Pig	*NKX3.2*: NC_010450	F: GAACCGTCGCTACAAGACCAA	qPCR
		R: CGGGAGGCAGTAATAAGGGT	
	GREM1: NC_010443	F: CATCATCAACCGCTTCTGCTAC	qPCR
		R: TCTTCTTCTTGGTGGGTGGC	
	WLS: NC_010448	F: GCGGAATCACATAGCAGGGTA	qPCR
		R: TGGACTGCTTCCCGCTGA	
	FGFR3: NC_010450	F: ATAGCCCGCACTACCTTTCA	qPCR
		R: GCCATGAGAACAGAGCCCTA	
	HHEX: NC_010456	F: CCCAACTCCTCCTTCACCAG	qPCR
		R: CAGGGCGTGCGTGTAGTCA	
	WNT16: NC_010460	F: ATCGCCTCCTTTGGGGTTC	qPCR
		R: TCGGCAGCAGGTACGGTTT	
	COL2A1: NC_010447	F: CAGCCAGCGTTCCCAAGA	qPCR
		R: AGTGGTAGGTGATGTTCTGGGAG	
	COL10A1: NC_010443	F: CCCACCCGAAGGCTTTGTA	qPCR
		R: TTTGGGTCATAGTGCTGTTGC	
	NKX3.2: NC_010443	F: TTCTGCTTTCCAGGATGCG	SqRT-PCR
		R: AATTCTGGGATTCAGGCTTT	
	β-actin: NC_010445	F: AGAGCAAGAGAGGCATCCTG	SqRT-PCR
		R: CACGCAGCTCGTTGTAGAAG	and qPCR
	ALDBSSCG0000004943	F: CGTTGGTTCCACGACTGCTA	qPCR
		R: CGTCTGTTAGCCTCCCGTTC	
	XLOC_074900	F: TAGGGTTCATTCTGCCGTCT	qPCR
		R: GCCTTCTCAAGCCAACTGC	
	XLOC_043998	F: TCGTGTCCTCTGGCTGTTCA	qPCR
		R: CTCAATGGTAGAGTCGGGTG	
	XLOC_007104	F: CAGAAGCACAGCAAGAAGGC	qPCR
		R: AAGGTGGGGAGCGAGGATAG	
	ALDBSSCG0000006642	F: TTCAGAAAGGCTCAATCCCC	qPCR
		R: CCCTTGCTCTTAGATGGTGCT	
	ALDBSSCG0000005264	F: GGCGTGCCTCCTAACATAA	qPCR
		R: CCTGGTGGGAAAACAGAAAT	
Mouse	*COL2A1*: NC_000081	F: GGAATTTGGTGTGGACATAGGG	qPCR
		R: GGTCAGGTCAGCCATTCAGT	
	COL10A1: NC_000076	F: CCTGGTTCATGGGATGTT	qPCR
		R: CCAGGAATGCCTTGTTCT	
	β-actin: NC_000071	F: GATGTGGATCAGCAAGCAGGA	qPCR
		R: AGGGTGTAAAACGCAGCTCAG	

Figure S1. Histological analysis of differences in chondrocytes size in TIC obtained from Y1, Y4, W1 and W4. Red arrow indicates proliferating chondrocytes, Green arrow indicates hypertrophic chondrocytes.

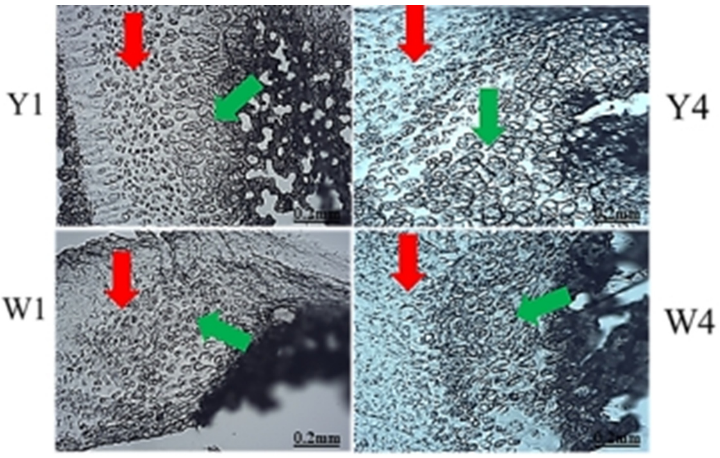

Figure S2. Pearson correlation coefficients of gene expression among the biological replicates from Y and W pigs.

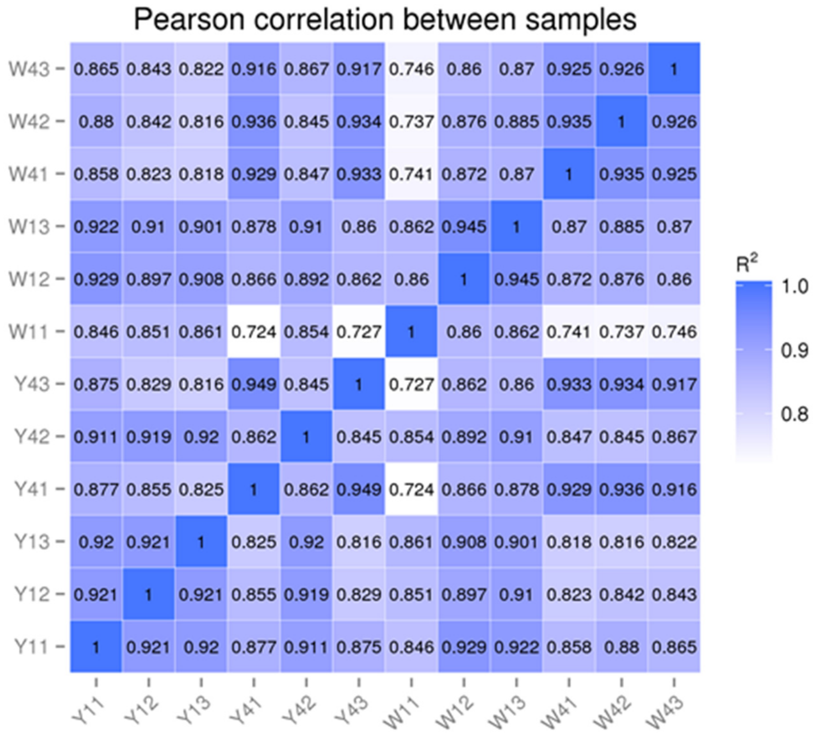

Figure S3. Identification lncRNAs in TIC transcriptome. (A) The workflow to select lncRNA. (B) Candidate lncRNAs were identified by using four applications: CNCI (coding-non-coding-index), CPC (coding potential calculator), PFAM-scan and PhyloCSF (phylogenetic codon substitution frequency) which detect and remove putative protein-coding transcripts.

A

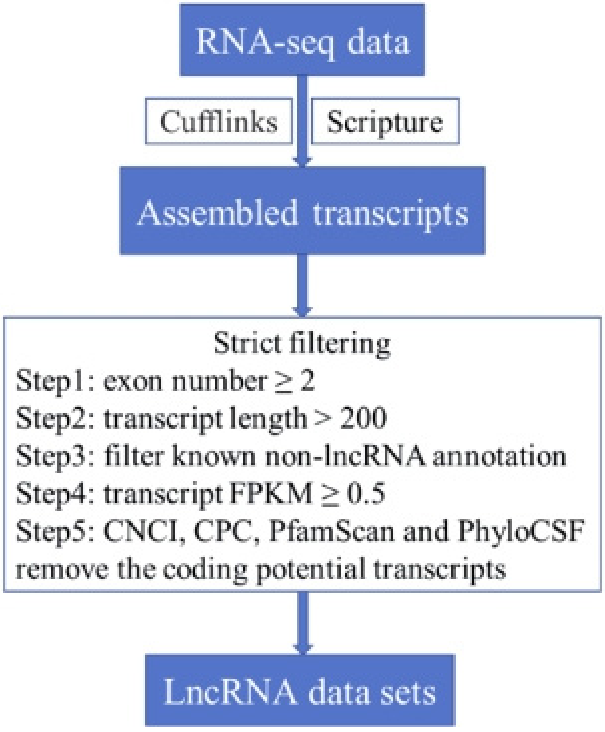

B

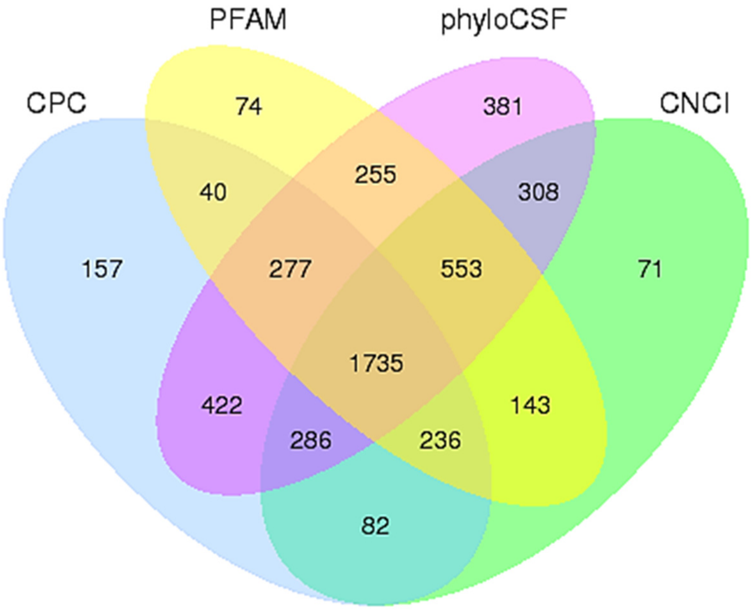

Figure S4. Venn diagrams of DE lncRNAs, DEGs and DE miRNAs showing Y4 vs. Y1 compared to W4 vs. W1 as well as Y4 vs. W4 compared to Y1 vs. W1. (A) Number of DE lncRNAs, DEGs and DE miRNAs for two comparisons of Y4 vs Y1 and W4 vs W1. (B) Number of DE lncRNAs, DEGs and DE miRNAs for two comparisons of Y4 vs W4 and Y1 vs W1.

A

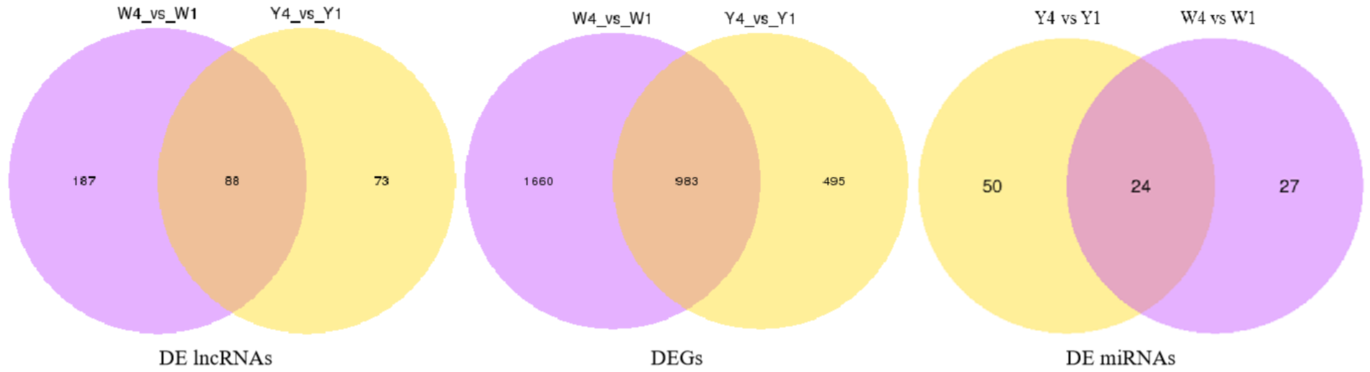

B

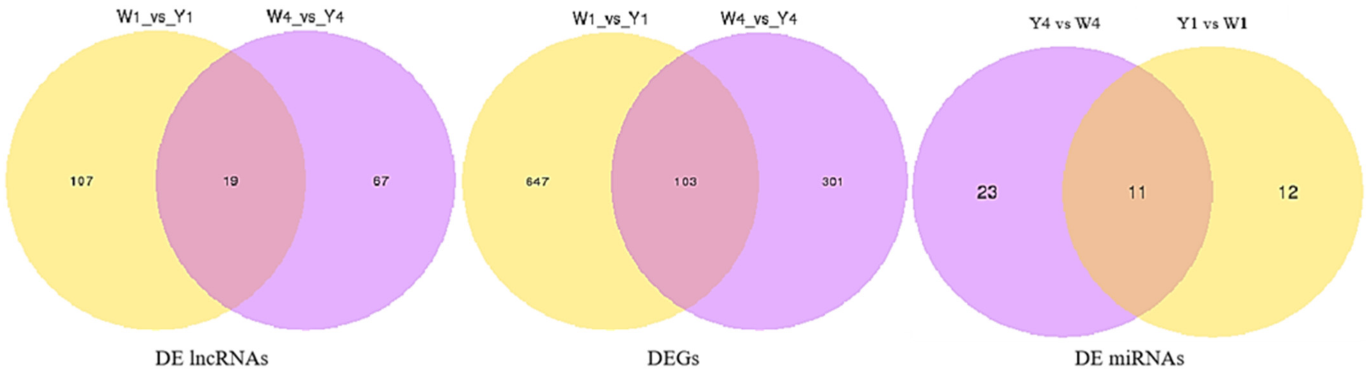

Figure S5. Crucial pathways were clustered from differentially expressed genes and noncoding RNAs target genes. GO terms analysis (A, C, E) and KEGG pathways (B, D, F) clustered by DEGs, DE lncRNAs and DE miRNAs, respectively.

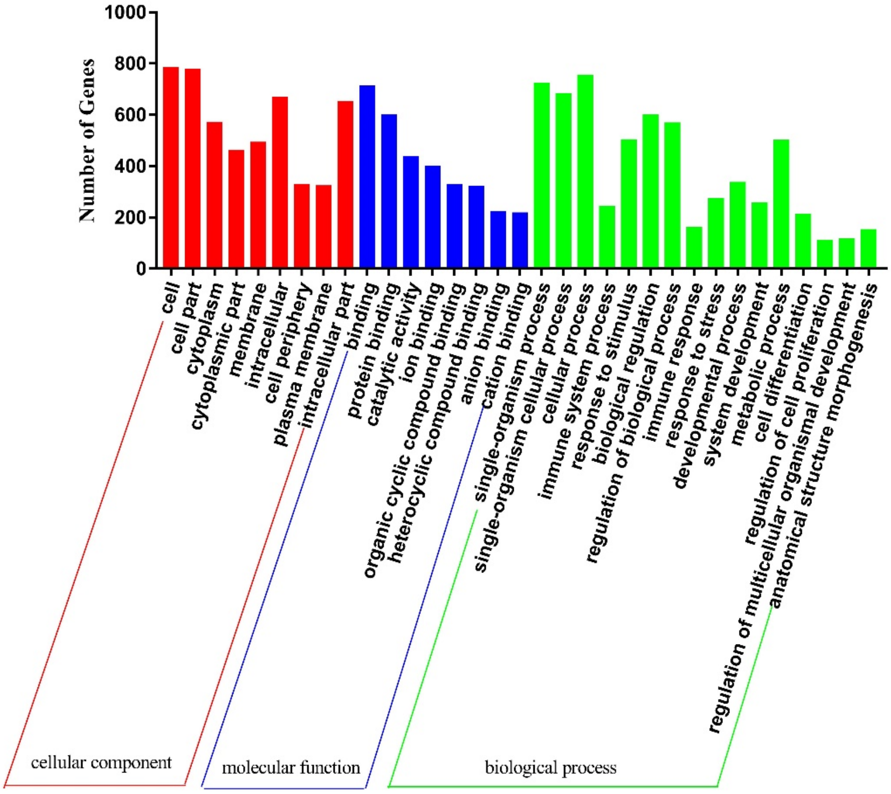

A

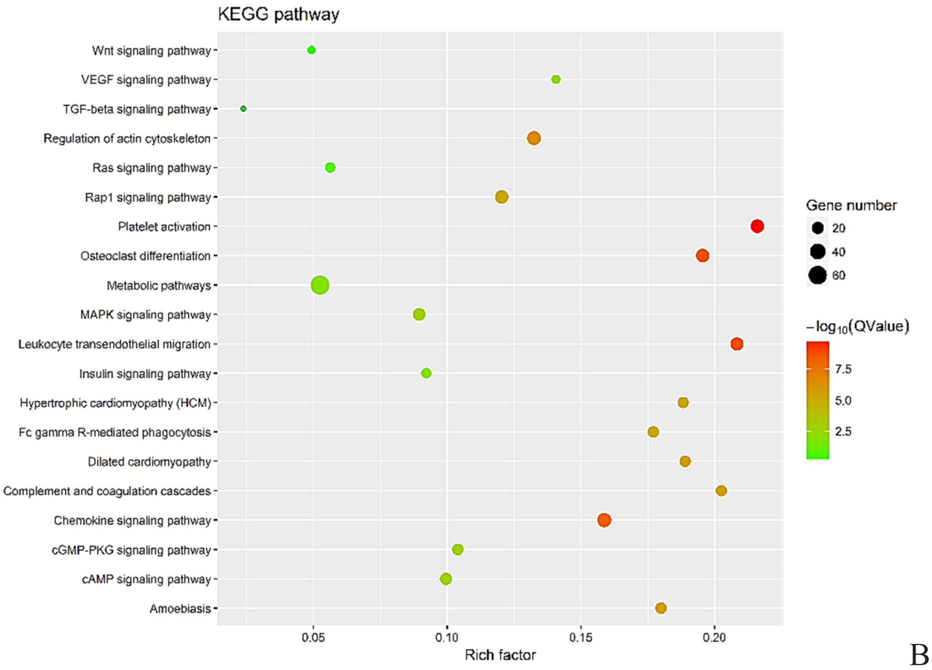

KEGG pathway
Wnt signaling pathway
VEGF signaling pathway
TGF-beta signaling pathway
Regulation of actin cytoskeleton
Ras signaling pathway
Rap1 signaling pathway
Platelet activation
Osteoclast differentiation
Metabolic pathways
MAPK signaling pathway
Leukocyte transendothelial migration
Insulin signaling pathway
Hypertrophic cardiomyopathy (HCM)
Fc gamma R-mediated phagocytosis
Dilated cardiomyopathy
Complement and coagulation cascades
Chemokine signaling pathway
cGMP-PKG signaling pathway
cAMP signaling pathway
Amoebiasis
0.05
0.10
0.15
0.20
Rich factor
Gene number
20
40
60
−log10(QValue)
7.5
5.0
2.5

B

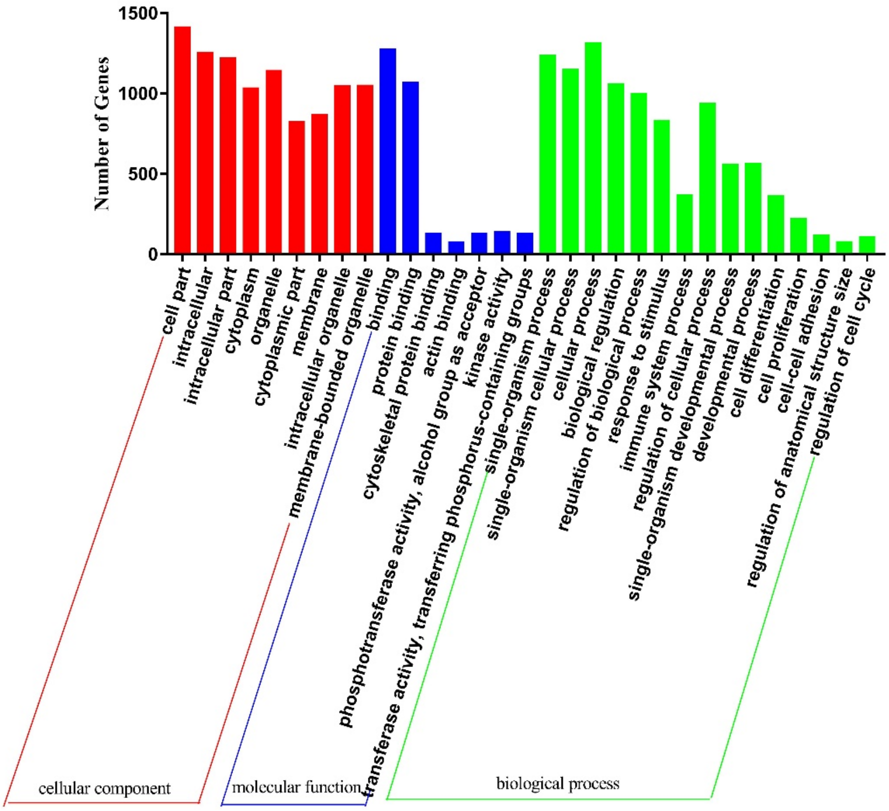
Number of Genes
1500
1000
500
0
cell part
intracellular
intracellular part
cytoplasm
organelle
cytoplasmic part
membrane
intracellular organelle
membrane-bounded organelle
binding
protein binding
cytoskeletal protein binding
actin binding
phosphotransferase activity, alcohol group as acceptor
kinase activity
transferase activity, transferring phosphorus-containing groups
single-organism process
single-organism cellular process
cellular process
biological regulation
regulation of biological process
response to stimulus
immune system process
regulation of cellular process
single-organism developmental process
developmental process
cell differentiation
cell proliferation
cell-cell adhesion
regulation of anatomical structure size
regulation of cell cycle
cellular component
molecular function
biological process

C

D

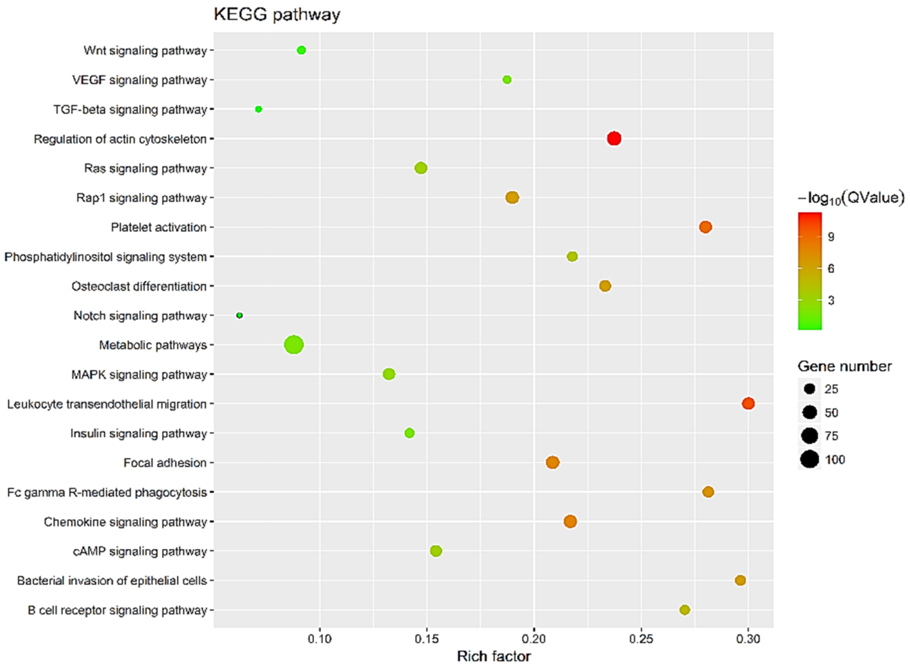
KEGG pathway
Wnt signaling pathway
VEGF signaling pathway
TGF-beta signaling pathway
Regulation of actin cytoskeleton
Ras signaling pathway
Rap1 signaling pathway
Platelet activation
Phosphatidylinositol signaling system
Osteoclast differentiation
Notch signaling pathway
Metabolic pathways
MAPK signaling pathway
Leukocyte transendothelial migration
Insulin signaling pathway
Focal adhesion
Fc gamma R-mediated phagocytosis
Chemokine signaling pathway
cAMP signaling pathway
Bacterial invasion of epithelial cells
B cell receptor signaling pathway
0.10
0.15
0.20
0.25
0.30
Rich factor
−log10(QValue)
9
6
3
Gene number
25
50
75
100

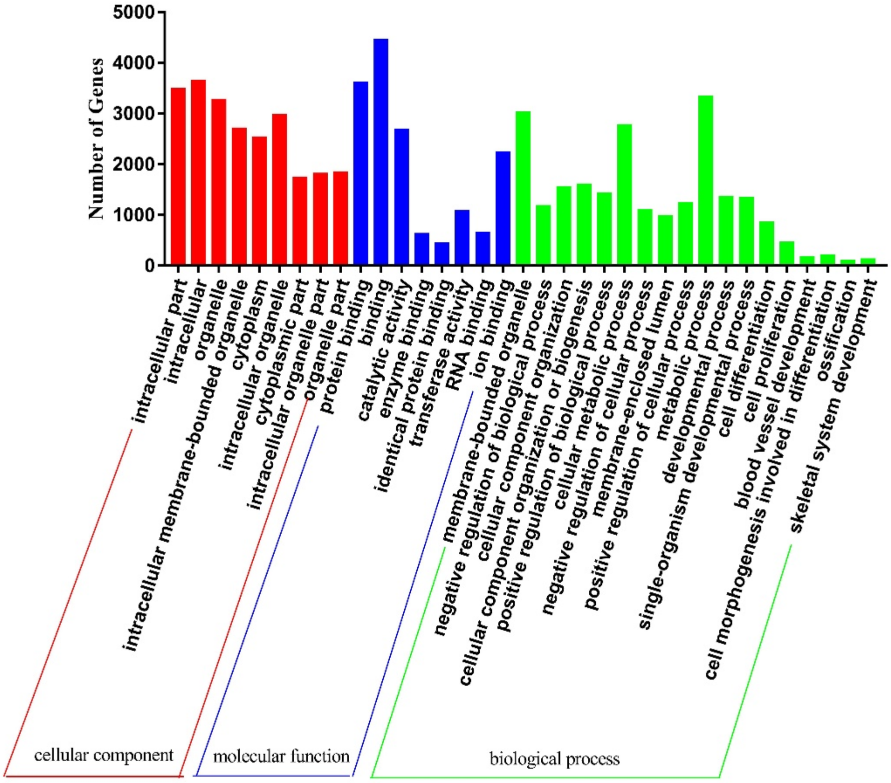
Number of Genes
5000
4000
3000
2000
1000
0
intracellular part
intracellular
organelle
intracellular membrane-bounded organelle
cytoplasm
intracellular organelle
cytoplasmic part
intracellular organelle part
organelle part
protein binding
binding
catalytic activity
enzyme binding
identical protein binding
transferase activity
RNA binding
ion binding
membrane-bounded organelle
negative regulation of biological process
cellular component organization
cellular component organization or biogenesis
positive regulation of biological process
cellular metabolic process
negative regulation of cellular process
membrane-enclosed lumen
positive regulation of cellular process
metabolic process
developmental process
single-organism developmental process
cell differentiation
cell proliferation
blood vessel development
cell morphogenesis involved in differentiation
ossification
skeletal system development
cellular component
molecular function
biological process

E

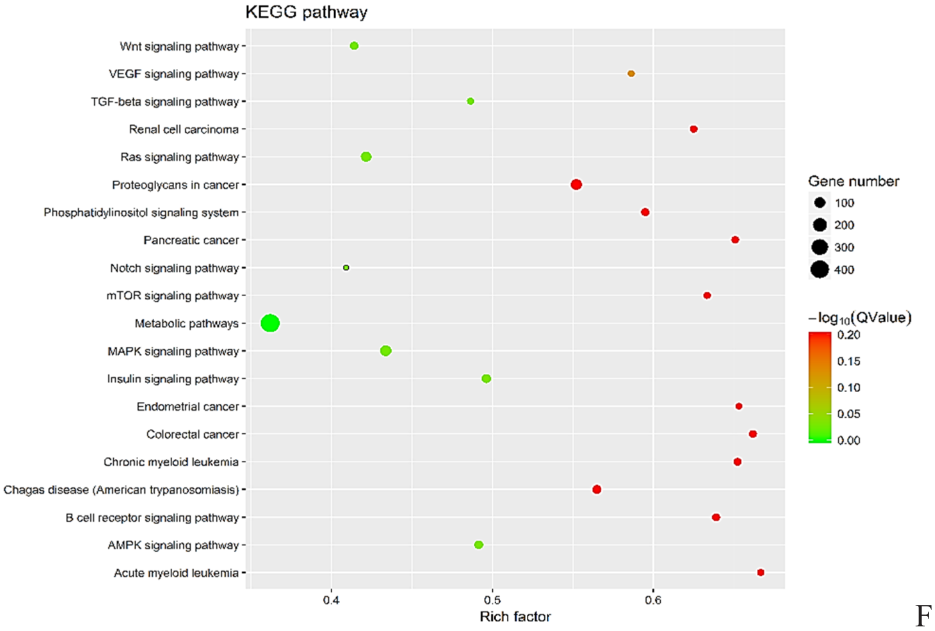
KEGG pathway
Wnt signaling pathway
VEGF signaling pathway
TGF-beta signaling pathway
Renal cell carcinoma
Ras signaling pathway
Proteoglycans in cancer
Phosphatidylinositol signaling system
Pancreatic cancer
Notch signaling pathway
mTOR signaling pathway
Metabolic pathways
MAPK signaling pathway
Insulin signaling pathway
Endometrial cancer
Colorectal cancer
Chronic myeloid leukemia
Chagas disease (American trypanosomiasis)
B cell receptor signaling pathway
AMPK signaling pathway
Acute myeloid leukemia
0.4
0.5
0.6
Rich factor
Gene number
100
200
300
400
−log10(QValue)
0.20
0.15
0.10
0.05
0.00

F
